# Molecular transmission network reveals CRF08_BC-driven clusters of HIV-1 among low-education older adults linked to female sex workers in Taizhou, China

**DOI:** 10.3389/fmicb.2026.1853569

**Published:** 2026-07-01

**Authors:** Junxiao Lin, Xiaolu Wang, Hongzhu Wang, Shanling Wang, Guixia Li, Yi Jiang, Tingting Wang, Yali Xie, Yating Wang, Jiafeng Zhang, Haijiang Lin

**Affiliations:** 1 Taizhou Municipal Center for Disease Control and Prevention, Taizhou Municipal Institute of Health Supervision, Taizhou, China; 2 Taizhou Municipal Jiaojiang District Center for Disease Control and Prevention, Taizhou Municipal Jiaojiang District Institute of Health Education, Taizhou, China; 3 Wenling Municipal Center for Disease Control and Prevention, Wenling Municipal Institute of Health Supervision, Taizhou, China; 4 Department of HIV/AIDS Control and Prevention, Zhejiang Provincial Center for Disease Control and Prevention, Hangzhou, China; 5 Zhejiang Key Laboratory of Vaccine, Infectious Disease Prevention and Control, Zhejiang Provincial Center for Disease Control and Prevention, Hangzhou, China

**Keywords:** CRF08_BC, female sex workers, HIV-1, molecular transmission network, older adults

## Abstract

**Background:**

The demographic structure of China’s HIV-1 epidemic is rapidly shifting toward an aging population. This study aims to analyze the molecular transmission network characteristics of newly diagnosed HIV-1 infected individuals aged 50 and above in Taizhou, China, by combining sociodemographic information.

**Methods:**

Plasma samples were collected from newly diagnosed HIV-1 patients aged ≥50 years who were not receiving antiretroviral therapy (ART) in Taizhou between 2023 and 2025. HIV-1 subtypes were determined and pairwise genetic distances were calculated based on the HIV-1 pol gene sequences amplified by nested PCR and sequenced by Sanger sequencing. A molecular transmission network was constructed using a genetic distance threshold of 0.010. Logistic regression was subsequently used to identify factors associated with molecular clustering.

**Results:**

Of the 506 samples, 473 (93.5%) were successfully sequenced. The predominant HIV-1 subtypes identified were CRF07_BC (34.0%), CRF08_BC (33.8%), and CRF01_AE (23.5%). Univariate analysis showed that molecular clustering was associated with marital status, education level, heterosexual transmission, and viral subtype. Subsequent multivariate analysis revealed that clustering was significantly correlated with heterosexual contact (aOR = 2.71, 95%CI: 1.46–5.25) and infection with either the CRF08_BC (aOR = 5.90, 95%CI: 3.39–10.46) or CRF07_BC (aOR = 1.85, 95%CI: 1.10–3.14) subtypes. Overall, 264 cases (55.8%) formed 64 distinct molecular clusters, comprising 6 large clusters and 58 small clusters. Notably, four of the six largest clusters belonged to the CRF08_BC subtype. Within the CRF08_BC clusters, 95.2% (120/126) of individuals had junior middle school education or lower, and female sex workers (FSWs) were identified in the two largest clusters. Additionally, a large cluster of 11 individuals comprising the minor subtype CRF85_BC was detected.

**Conclusion:**

Molecular transmission networks and sociodemographic data indicate that older, less-educated individuals are facilitating the spread of the HIV-1 CRF08_BC subtype in Taizhou via contact with FSWs. Therefore, targeted interventions focusing on elderly men with low education levels are essential.

## 1 Introduction

According to the 2025 Global AIDS Update of the Joint United Nations Programme on HIV/AIDS (UNAIDS), by the end of 2024, an estimated 40.8 million people worldwide was projected to be infected with HIV (UNAIDS, 2025), and the prevalence of human immunodeficiency virus (HIV) remains a major global public health challenge. As antiretroviral treatment has been widely implemented, which has greatly extended the life span of infected people, the age structure of HIV-1 infected people has turned to aging (Brooks et al., 2012; Autenrieth et al., 2018). Similarly, this phenomenon is also reflected in China (Chen et al., 2012; Xing et al., 2014). In recent years, the number of newly diagnosed infections among adults aged 50 and above in China has continued to increase each year, rising from 11,751 in 2010 to 51,856 in 2022 (Hou et al., 2023), posing significant challenges to regional disease control. Relying solely on traditional epidemiological surveys has obvious limitations for this specific group. Research has shown that the elderly population still maintains a certain level of sexual activity (Lindau et al., 2007; Wang, B. et al., 2023), but due to recall bias or shame, self-report is often inaccurate (Emlet et al., 2015), thus masking the true transmission pathway (Sun et al., 2022).

In order to compensate for the data loss caused by traditional epidemiological investigations, molecular transmission networks (MTNs) provide a new methodological path (Wertheim et al., 2017). MTNs can reconstruct the viral transmission network with higher accuracy by conducting in-depth analysis of viral genome sequences and phylogenetic data (Kosakovsky Pond et al., 2018). With the help of sequence analysis technology, researchers can not only uncover hidden transmission associations and accurately locate transmission clusters, but also dynamically track the evolution trajectory of the epidemic (Ragonnet-Cronin et al., 2019; Novitsky et al., 2021), providing powerful data supplements for traditional contact tracing and epidemiological investigations (Zhang, J. et al., 2023).

Research has shown that older adults play a significant role in maintaining regional HIV-1 MTNs (Ma et al., 2021). At the same time, studies have found that patients aged 50 and above tend to have stronger clustering and are more likely to appear in transmission clusters with the mainstream HIV-1 subtype (Fan et al., 2025). Research on female sex workers (FSWs) also reflects the complexity of dynamic transmission within populations (Zhang, L. et al., 2015; Springfield et al., 2023). However, despite these preliminary observations, most MTN studies have focused predominantly on younger populations and traditionally high-risk groups, such as men who have sex with men. Consequently, the molecular transmission patterns and structural drivers among older adults, as well as the extent to which sociodemographic factors interact with viral subtypes within local networks, remain insufficiently understood. To address this gap, this study aimed to characterize HIV-1 MTNs among individuals aged 50 years and older and to identify key factors associated with clustered transmission, providing evidence to inform more targeted and precise public health interventions.

## 2 Materials and methods

### 2.1 Study population

This study included 506 newly diagnosed HIV-1 infected individuals aged 50 and above in Taizhou from 2023 to 2025, and collected their plasma samples. All selected individuals did not receive antiretroviral therapy at the time of diagnosis. The basic socio demographic information and clinical data of patients, including gender, age, education level, marital status, transmission routes, and the first CD4 + T cell counts, were all obtained from the surveillance database of Taizhou Municipal Center for Disease Control and Prevention, Taizhou Municipal Institute of Health Supervision. This study was approved by the Institutional Ethics Committee of Taizhou Municipal Center for Disease Control and Prevention, Taizhou Municipal Institute of Health Supervision (Approval No. TZCDC-2025-11R-011).

### 2.2 Experimental operation

The whole blood sample is processed by the Taizhou Municipal Center for Disease Control and Prevention. After centrifuging the sample 10 min at 3000 rpm, the separated plasma was collected and store it at −80 °C. The HIV-1 LAg-Avidity enzyme-linked immunosorbent assay kit (Kinghawk, Beijing, China) was used to distinguish recent infection from established infection for surveillance purposes. According to the kit instructions, a threshold of normalized optical density (ODn) ≤1.5 is used to classify recent infections. This threshold corresponds to a mean duration of recent infection (MDRI) of approximately 130 days.

Viral RNA was extracted from plasma using the RNA Extraction Kit (Tianlong, Suzhou, China). By reverse transcription polymerase chain reaction and subsequent nested polymerase chain reaction, the target fragment on HIV-1 pol gene was amplified, which covers the full-length protease and the first 300 amino acids of reverse transcriptase (corresponding to HXB2 coordinates 2,147–3,462; Zhang, J. et al., 2015). The positive amplification product obtained was sent to Hangzhou Qingke Biotechnology Co., Ltd. for purification and Sanger sequencing.

### 2.3 Sequence analysis and subtype determination

The original gene sequences were assembled and manually corrected using Sequencher v5.4.6 (Gene Coding Company, Ann Arbor, Michigan, USA), followed by quality assessment and multiple sequence alignment using BioEdit v7.0.9. In terms of subtype identification, reference sequences were extracted from the HIV sequence database at Los Alamos National Laboratory as controls. To screen for potential recombination events, sequences were further analyzed using the LANL Recombinant Identification Program (RIP) and SimPlot v3.5.1 (Han et al., 2013). Subsequently, IQ-TREE v2.3.6 software was used to construct a maximum likelihood phylogenetic tree based on the GTR + G + I nucleotide substitution model. The robustness of phylogenetic clustering was assessed using 1,000 bootstrap replicates. Bootstrap support values greater than 70% were considered to indicate moderate-to-strong support for phylogenetic clusters (Zhang, J. et al., 2015). Node support was further evaluated using the SH-like approximate likelihood ratio test to provide additional confidence for tree topology. The final constructed maximum likelihood phylogenetic tree was visualized using the iTOL v7.5 online platform.

### 2.4 Construction and analysis of molecular transmission network

In this study, consistent with previous studies (Wertheim et al., 2017; Kosakovsky Pond et al., 2018; Oster et al., 2018; Liu et al., 2020; Rich et al., 2023; Wang, Z. et al., 2023; Chinese Center for Disease Control and Prevention, 2025), molecular transmission networks were constructed based on a genetic distance approach. Using MEGA v.6.06 software and based on the Tamura-Nei 93 evolutionary model, the pairwise genetic distance (GD) between each sequence was calculated. Within the range of 0.001 to 0.020, the clustering effect was systematically evaluated at different genetic distance thresholds with a step size of 0.001. A genetic distance threshold of 0.010 was chosen as the genetic distance threshold, at which the dataset identified the highest number of molecular clusters. A molecular transmission cluster was defined by a maximum intra-cluster pairwise genetic distance of ≤0.010. The visualization of molecular networks was completed through Cytoscape v3.10.4. In a molecular network, each node represents a patient, the potential transmission relationships are denoted by connecting edges (links). The node degree (number of edges) reflects its transmission potential and centrality in the network. Based on cluster size, the identified molecular clusters are divided into small clusters (SCs, <10 nodes) and large clusters (LCs, ≥10 nodes).

### 2.5 Statistical analysis

All statistical analysis and graphical visualizations were completed using R software (version 4.5.2). Baseline demographic and clinical characteristics were used as categorical variables and described in terms of frequency and percentage. To screen for factors that are independently associated with clustering, univariable analysis was first used for analysis. Variables with *p*-values less than 0.05 in the single factor analysis were included in the multivariate analysis to control for the influence of confounding factors. In addition, to reveal the structural characteristics of the molecular network, a bar chart was used to show the proportional distribution of different HIV-1 subtypes in terms of cluster size, link, and education level. Statistical significance is defined as a two-sided *p* value less than 0.05.

## 3 Results

### 3.1 Distribution of HIV-1 subtypes

**Figure 1:**
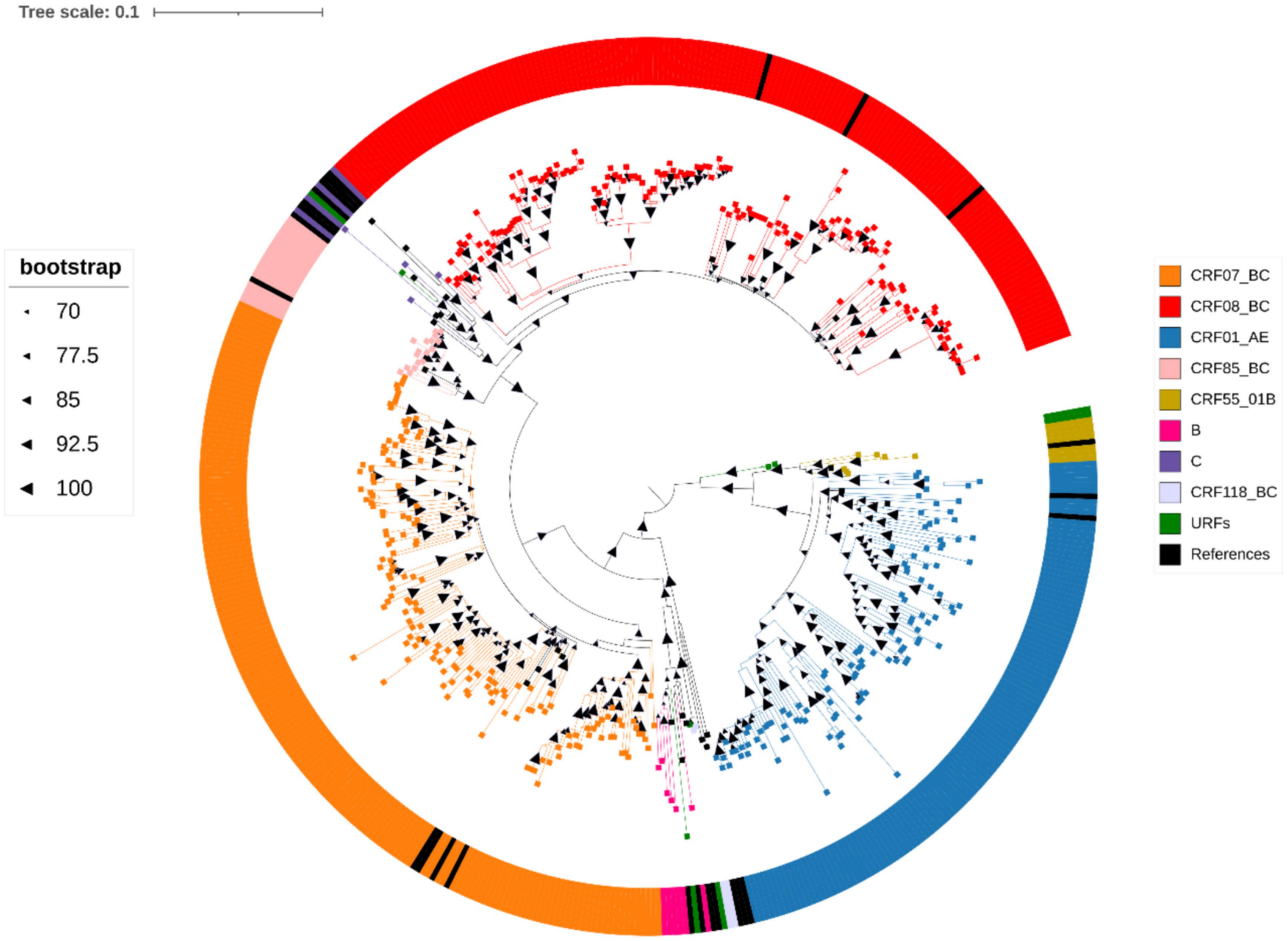
Maximum likelihood phylogenetic tree of HIV-1 pol sequences from newly diagnosed cases aged ≥50 years in Taizhou, China, 2023–2025. Colors denote subtypes.

Between 2023 and 2025, 506 newly diagnosed HIV-1 cases aged ≥50 years were enrolled in Taizhou, of whom 473 (93.5%) were successfully amplified and yielded the desired pol gene sequences. After conducting phylogenetic analysis on the above gene sequences, it was found that the local HIV-1 strains exhibit high diversity, covering 8 different subtypes and a few unique recombinant forms (URFs). The proportions of CRF07_BC and CRF08_BC are similar, accounting for 34.0% (161/473) and 33.8% (160/473) respectively, while CRF01_AE accounts for 23.5% (111/473). The remaining subtypes are CRF85_BC (3.6%, 17/473), CRF55_01B (1.5%, 7/473), B subtype (1.3%, 6/473), C subtype (0.8%, 4/473), CRF118_BC (0.4%, 2/473), and URFs (1.1%, 5/473; Figure. 1).

### 3.2 Demographic characteristics of clustered patients

Among 473 patients aged 50 and above, 264 individuals (55.8%) were included in the molecular transmission network. Univariate analysis results indicated significant associations between marital status, education level, transmission route, and HIV-1 subtypes with clustering (Table 1).

**Table 1:** Demographic characteristics of clustered patients (aged ≥50) in Taizhou, China, 2023–2025.

Variables	Total	Number of clustered *N* (%)	Univariate analysis		Multivariate analysis	
			OR (95% CI)	*p* value	aOR (95% CI)	*p* value
Gender						
Male	349	191 (54.7)	1.00 (ref)	–		
Female	124	73 (58.9)	1.18 (0.78–1.80)	0.425		
Marital status						
Married	296	179 (60.5)	1.00 (ref)	–	1.00 (ref)	–
Divorced/widowed	141	71 (50.4)	0.66 (0.44–0.99)	0.046	0.68 (0.43–1.07)	0.095
Single	34	14 (41.2)	0.46 (0.22–0.94)	0.034	0.47 (0.21–1.01)	0.054
Unknown	2	0 (0.0)	-	0.981		
Education level						
Primary school/illiterate	311	187 (60.1)	1.00 (ref)	–	1.00 (ref)	–
Junior middle school	133	61 (45.9)	0.56 (0.37–0.85)	0.006	0.65 (0.41–1.03)	0.067
Senior high school	22	12 (54.5)	0.80 (0.33–1.94)	0.606	0.67 (0.26–1.76)	0.410
College or above	7	4 (57.1)	0.88 (0.19–4.55)	0.873	1.09 (0.21–6.22)	0.916
Transmission route						
Homosexual	63	16 (25.4)	1.00 (ref)	–	1.00 (ref)	–
Heterosexual	410	248 (60.5)	4.50 (2.52–8.43)	<0.001	2.71 (1.46–5.25)	0.002
The first CD4 + T cells (cells/μl)						
≥200	253	150 (59.3)	1.00 (ref)	–		
<200	220	114 (51.8)	0.74 (0.51–1.06)	0.103		
HIV-1 subtypes						
CRF01_AE	111	39 (35.1)	1.00 (ref)	–	1.00 (ref)	–
CRF08_BC	160	126 (78.8)	6.84 (4.01–11.92)	<0.001	5.90 (3.39–10.46)	<0.001
CRF07_BC	161	79 (49.1)	1.78 (1.09–2.94)	0.023	1.85 (1.10–3.14)	0.021
Others	41	20 (48.8)	1.76 (0.85–3.65)	0.128	1.78 (0.84–3.80)	0.132

OR, odds ratio; aOR, adjusted odds ratio; CI, confidence interval.

After further adjusting for potential confounding factors using a multivariable logistic regression model, it was found that patients with heterosexual transmission had a higher clustering probability compared to those with homosexual transmission (aOR = 2.71, 95% confidence interval: 1.46–5.25, *p* = 0.002). HIV-1 subtypes also served as independent predictors: patients infected with CRF08_BC (aOR = 5.90, *p* < 0.001) or CRF07_BC (aOR = 1.85, *p* = 0.021) had a higher clustering probability than those infected with CRF01_AE.

**Figure 2:**
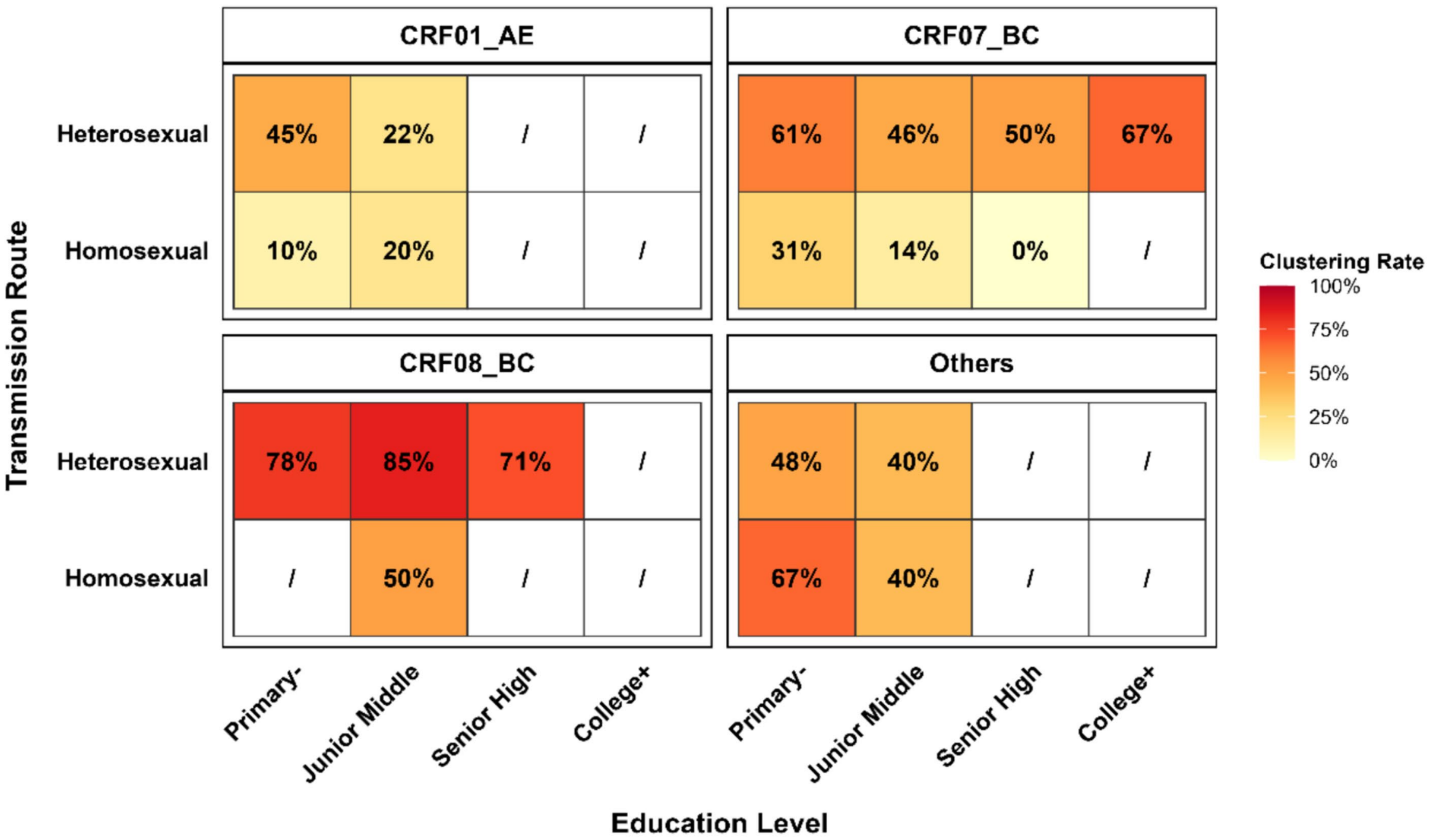
Heatmap of HIV-1 clustering rates stratified by subtype, transmission route, and education level.

By plotting a multi-dimensional risk heatmap (Figure 2), the results indicate that patients with characteristics such as heterosexual transmission, infection with CRF08_BC, and educational level of junior middle school or below had a very high clustering rate, reaching up to 85%. In contrast, unclustered cases are more common among unmarried or highly educated individuals. These findings suggest that among individuals aged ≥50 years, HIV-1 molecular transmission network dynamics are driven by the interplay of specific behavioral risk factors and circulating viral strains, rather than by a single determinant.

### 3.3 Characteristics of the HIV-1 MTN and subtype distribution

**Figure 3:**
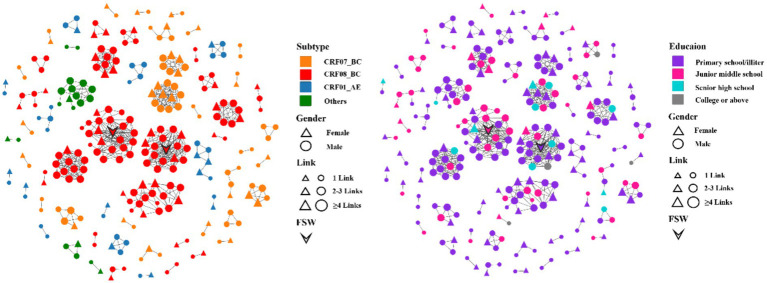
Molecular transmission network of HIV-1 infections among individuals aged ≥50 years in Taizhou. Nodes represent patients, and edges represent putative transmission relationships with pairwise genetic distance (GD) ≤0.010.

A total of 264 HIV-1 sequences were successfully clustered into 64 clusters (Figure 3). The entire MTN structure can be divided into 58 small clusters and 6 large clusters. Analysis at the subtype level reveals significant differences in the cluster sizes formed by different subtypes within MTN. CRF08_BC exhibited the highest clustering rate; 126 samples formed 20 clusters, comprising the four largest clusters in the molecular transmission network and 16 small clusters. CRF07_BC sequences (*n* = 79) were distributed across 26 molecular clusters, comprising one large cluster (the fifth largest) and 25 small clusters. In contrast, all 39 samples of CRF01_AE form 13 small clusters. Among the minor subtypes, CRF85_BC forms a large cluster containing 11 nodes. Analysis at the education level showed that of the 264 clustered samples, 248 (93.9%) had an education level of junior middle school or below.

**Figure 4:**
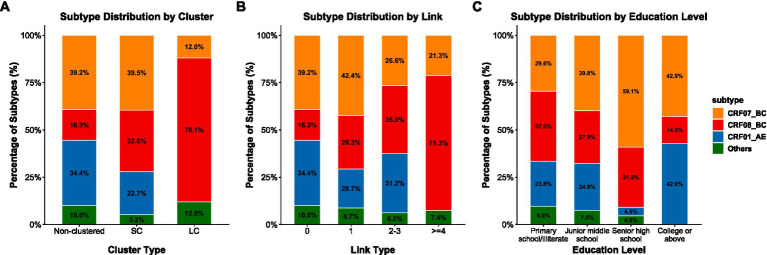
**(A)** Subtype distribution categorized by cluster scale: Non-clustered cases, small clusters (SC, <10 nodes), and large clusters (LC, ≥10 nodes). **(B)** Subtype distribution based on individual node degree (link counts ranging from 0 to ≥4). **(C)** Subtype distribution stratified by the highest level of formal education achieved by the clustered individuals.

After further analysis of subtypes based on cluster size and node degree, it was found that the structural advantage of CRF08_BC in MTN was further highlighted. From the scale of clustering, CRF07_BC and CRF01_AE mainly appear in non-clustered cases and small clusters, while at the large cluster level, the proportion of CRF08_BC is as high as 76.1% (Figure 4A). Similar trends are also reflected in node degree: as the degree of nodes increases, the proportion of CRF08_BC infected individuals gradually increases, reaching 71.3% in highly connected core nodes with four or more links (Figure 4B). Furthermore, within the two largest CRF08_BC clusters, some FSWs exhibited high network connectivity, with degrees of 13 and 15, respectively. This feature suggests that commercial sexual activity may serve as a bridge to promote the expansion of MTN, driving the widespread spread of CRF08_BC among people aged 50 and above. In terms of education level (Figure 4C), among the group with the lowest education level (primary school or illiterate), the proportion of CRF08_BC is the highest, reaching 39.8%. These findings identify a risk profile: low education level, involvement in commercial sexual activities, and infection with the CRF08_BC subtype are key factors associated with HIV-1 infection among individuals aged 50 and above in Taizhou.

### 3.4 Characteristic analysis of the six large clusters

Feature analysis was conducted on six large clusters (LC1-LC6), and their main features are summarized in Table 2. The size of these six clusters ranges from 11 to 23 nodes. From the distribution of subtypes, CRF08_BC constitutes the four largest clusters (LC1, 23 nodes; LC2, 18 nodes; LC3, 17 nodes; LC4, 12 nodes), while LC5 and LC6 (both 11 nodes) are composed of CRF07_BC and CRF85_BC, respectively.

**Table 2:** Summary characteristics of six LCs in Taizhou from 2023 to 2025.

LC	Subtype	Nodes	Diagnosis year (%)			Junior middle school or below (%)	Heterosexual (%)	Recent HIV-1 infection (%)	Taizhou household registration (%)
			2023	2024	2025				
LC1	CRF08_BC	23	6 (26.1)	7 (30.4)	10 (43.5)	22 (95.7)	21 (91.3)	4 (17.4)	23 (100.0)
LC2	CRF08_BC	18	11 (61.1)	3 (16.7)	4 (22.2)	15 (83.3)	18 (100.0)	1 (5.6)	10 (55.6)
LC3	CRF08_BC	17	9 (52.9)	5 (29.4)	3 (17.7)	17 (100.0)	17 (100.0)	2 (11.8)	17 (100.0)
LC4	CRF08_BC	12	1 (8.3)	10 (83.4)	1 (8.3)	11 (91.7)	12 (100)	3 (25.0)	7 (58.3)
LC5	CRF07_BC	11	3 (27.3)	4 (36.4)	4 (36.4)	8 (72.7)	11 (100.0)	4 (36.4)	11 (100.0)
LC6	CRF85_BC	11	3 (27.3)	5 (45.4)	3 (27.3)	10 (90.9)	11 (100)	2 (18.2)	7 (63.6)

The time distribution by diagnosis year shows that between 2023 and 2025, the number of nodes in the six LCs continues to increase, indicating ongoing active transmission. In terms of education level, low education level is a common feature—in each large cluster, the majority of people have a junior middle school education or below, ranging from 72.7% in LC5 to 100.0% in LC3. In terms of transmission routes, heterosexual contact is the dominant mode of transmission, accounting for 91.3% in LC1 and 100.0% in the other five major clusters (LC2-LC6).

LAg-Avidity EIA-based recent infection testing revealed that the proportion of recently infected individuals within each cluster ranged from 5.6% (LC2) to 36.4% (LC5). Specifically, these recent infections were predominantly concentrated in 2024–2025. LC1 contained 4 recent infections (1 in 2024 and 3 in 2025); LC3 and LC4 each contained 2 (both in 2024). Additionally, LC5 contained 2 (1 in 2024 and 1 in 2025), and LC6 contained 1 (2024), indicating differential transmission activity across clusters. Regarding registered residence, all patients in LC1, LC3, and LC5 are locally registered in Taizhou, while local residents account for 55.6–63.6% of patients in LC2, LC4, and LC6, with a more heterogeneous composition.

## 4 Discussion

Historically, older HIV-1 infected individuals have often been perceived as terminal nodes in transmission chains due to recall bias and social stigma (Curley and Johnson, 2022). However, by combining sociodemographic information with molecular transmission network (MTN) analysis (Xu et al., 2024), we obtained strong evidence of active, hidden transmission in the older adult population (≥50 years) of Taizhou City. We found a clustering rate of 55.8% in this population, significantly higher than the national average for this population. This suggests that older adults are not merely passive recipients of the virus, but active and key players in maintaining and expanding regional HIV-1 transmission networks (Wu et al., 2019; Jiang et al., 2020).

A key finding of this research is that HIV-1 subtypes exhibit significant differences in the MTN within older adults (Vrancken et al., 2020; Zhang, F. et al., 2023). Although the ratio of CRF07_BC to CRF08_BC is almost the same, the latter exhibits significantly stronger clustering ability, forming the four largest and most complex transmission clusters. The advantages of CRF08_BC in the MTN reflect its strong biological adaptability and transmission efficiency in the context of heterosexual transmission (Feng et al., 2013; Han et al., 2013). In addition, we observed an 11-node cluster composed of a novel recombinant CRF85_BC, indicating that the diversity of HIV-1 strains in local areas is still evolving (Hassan et al., 2018; Liu et al., 2020). While CRF85_BC is not the locally dominant subtype, it is primarily reported in southwestern regions such as Sichuan and Yunnan (Su et al., 2020; Liu et al., 2025). Notably, this cluster includes two patients registered in Sichuan and Yunnan, respectively. Additionally, it includes a patient newly infected in 2024. These findings suggest that this recombinant strain may have cross-regional transmission links, highlighting its ability to spread covertly and independently form a large cluster. Furthermore, combined with observations in other clusters, these recent infections concentrated in 2024–2025 strongly suggest that the aforementioned transmission networks still pose a high risk of triggering sustained local transmission at this stage. Therefore, in order to translate these findings into actionable disease control measures, future public health interventions need to shift toward more proactive and sustained genetic surveillance strategies. Our study highlights the innovative necessity of targeted active surveillance, which not only require accurate tracking of large, active transmission clusters that have triggered local outbreaks, but more importantly, require maintaining high vigilance to prevent the covert spread of secondary epidemic subtypes before they develop into self-sustaining large clusters (Yebra et al., 2018). It should be noted that because this study only included infected individuals aged ≥50 years, it was unable to analyze the transmission associations between this older adult population and other age groups. Excluded cases under 50 years of age may act as “bridge nodes” connecting different older adult cases, potentially leading to an underestimation of the number and size of existing molecular clusters.

Combining MTN analysis with sociodemographic data (Wang, Z. et al., 2023) revealed a unique transmission risk pattern significantly influenced by educational background. Specifically, patients with primary school education or below not only had a significantly higher rate of CRF08_BC infection but also occupied a prominent position in the largest transmission clusters. Female sex workers (FSWs) further exacerbated this risk, playing a key connecting role in these large clusters. Due to their low level of education, insufficient awareness of safe sex, and limited access to AIDS prevention resources, coupled with continued sexual activity, the elderly are driven into unprotected commercial sex networks (Mitchell et al., 2013). In this context, FSWs function as key connecting nodes, accelerating HIV-1 dissemination among the less educated elderly population.

## Limitations

There are several limitations to this study: First, self-reported sociodemographic data are inherently subject to recall bias and social stigma, which may lead to underestimation of the true involvement of FSWs; however, molecular transmission network analysis provided objective insights that complemented these data. Second, strict inclusion criteria limited the analysis to high-quality pol sequences from newly diagnosed patients, thereby excluding undiagnosed individuals or those with low viral loads. This may result in incomplete network representation, although the observed clustering rate suggests capture of major local transmission epidemic (Rich et al., 2023). Third, this study only included individuals aged 50 and older; therefore, the constructed network may not fully capture the overall local HIV-1 transmission dynamics. Although our current findings adequately reflect transmission patterns within the elderly population, future studies should place this population within the broader population-wide epidemiological context to further clarify their role and contribution to local epidemic dynamics.

## Conclusion

In summary, by combining molecular transmission networks (MTNs) with sociodemographic characteristics, this study revealed the local transmission dynamics of HIV-1 in the elderly population aged 50 and above in Taizhou. The results indicate that this population not only has an extremely high clustering rate but also plays a crucial role in maintaining regional transmission networks. Although the overall prevalence of CRF07_BC and CRF08_BC is comparable, CRF08_BC exhibits a clear structural advantage in driving large, complex transmission clusters. Low education levels and heterosexual transmission (particularly the bridging role of FSWs) are the core driving forces behind the widespread expansion of this dominant subtype. Furthermore, the large, active transmission clusters initiated by the non-dominant subtype CRF85_BC further highlight the ongoing evolution of the HIV-1 strain pool and the risk of covert transmission in this region. These findings underscore the importance of implementing precise genomic surveillance targeting elderly men with low educational backgrounds and related commercial sex networks.

## Data Availability

The datasets presented in this study can be found in online repositories. The names of the repository/repositories and accession number(s) can be found at: https://ngdc.cncb.ac.cn/genbase, C_AA167588.1 - C_AA168060.1.
